# Nutritional and Functional Effects of *Ganoderma lucidum*-Fermented Mulberry Leaves in Aged Laying Hens

**DOI:** 10.3390/ani16172697

**Published:** 2026-08-31

**Authors:** Zhenzhen Liu, Qiqi Chen, Xiaosen Bai, Shuzhen Zhao, Xiaoxuan Bai, Jianyang Shen, Dandan Hu, Zhiwei Zhang, Zhiding Guo, Xiaoli Xu, Yang Song, Xiaobin Feng

**Affiliations:** 1 Cangzhou Academy of Agriculture and Forestry Sciences, Cangzhou 061001, China; 2 Institute of Food Science Technology Nutrition and Health (Cangzhou), Chinese Academy of Agricultural Sciences, Cangzhou 061000, China; 3 Nanpi County Agriculture and Rural Affairs Bureau, Cangzhou 061500, China

**Keywords:** *Ganoderma lucidum*, mulberry leaf, solid-state fermentation, laying hens, serum biochemical profile

## Abstract

Solid-state fermentation (SSF) can reduce antinutritional factors and improve the nutritional value of plant feed substrates. Here, we characterized mulberry leaves fermented with *Ganoderma lucidum* and evaluated a 3% dietary inclusion level in aged laying hens. Fermentation increased crude protein by 19.51% and increased total, essential, and branched-chain amino acids. Tannin and phytic acid contents decreased by 31.03% and 19.61%, respectively. After 60 days, the FML group showed a laying rate of 76%, comparable to Con (76%) and UML (74%), and no significant differences were detected in egg quality traits; however, the FML diet significantly reduced egg-yolk cholesterol relative to both Con and UML. Both UML and FML increased serum T-AOC, SOD, and CAT activities and reduced MDA. FML produced greater increases in T-AOC and SOD than UML. UML increased serum IgA, whereas FML increased IgG. Both products reduced serum TC and LDL-C. Thus, *G. lucidum*-mediated SSF improved the nutritional profile of mulberry leaves and altered selected physiological indices at a 3% dietary inclusion level. Dose–response and mechanistic studies are needed to establish efficacy and practical value. The mechanisms underlying the observed changes in antioxidant status, serum lipid profiles, and immunoglobulin concentrations require further elucidation.

## Simple Summary

Aged hens are more prone to oxidative stress, disrupted lipid metabolism, and reduced laying performance. This study examined whether solid-state bioprocessing by *Ganoderma lucidum* enhances the nutritional quality of mulberry leaves and improves their suitability as a functional feed ingredient for aged laying hens. Fermentation increased crude protein and most amino acids while reducing tannins and phytic acid. Feeding 3% fermented mulberry leaf powder did not impair laying performance or conventional egg-quality traits. It reduced egg-yolk cholesterol and improved several serum antioxidant, lipid-metabolism, and immunoglobulin indices. These findings indicate that *G. lucidum*-fermented mulberry leaves may be useful at a low dietary inclusion level. However, dose–response studies and mechanistic analyses are required before commercial recommendations can be made.

## 1. Introduction

Eggs represent a major source of high-quality animal protein, and global demand continues to rise [1]. This growth places pressure on the laying-hen industry to sustain productivity, egg quality, and bird health throughout the production cycle. Ageing hens are increasingly susceptible to impaired intestinal function, oxidative stress, and altered lipid metabolism [2]. These changes can coincide with lower laying rates and reduced eggshell quality during the late laying phase [3,4]. Nutritional strategies based on functional feed ingredients may therefore help maintain late-cycle production and health.

Mulberry leaves (*Morus alba* L.) contain proteins, soluble sugars, organic acids, flavonoids, polyphenols, amino acids, and trace elements. They have a long history of use in traditional Chinese medicine and are now receiving growing attention as potential functional feed ingredients [5,6]. Mulberry-derived products can modulate glucose and lipid metabolism in experimental models [7,8]. In poultry, mulberry leaf supplementation has reduced abdominal fat deposition in broilers [9], improved antioxidant status in laying hens [10], and reduced hepatic lipid accumulation and egg-yolk cholesterol-related indices [11,12]. However, tannins and phytic acid can limit nutrient utilization when their concentrations are not controlled [13,14,15].

Fermentation processes, including SSF, are known to reduce antinutritional compounds and improve the nutritional quality, digestibility, and bioavailability of plant-based substrates [16,17,18]. In laying hens, fermented feed has been reported to enhance egg quality, immune indices, intestinal structure, and microbial composition during the late production cycle [19,20]. Moreover, recent evidence indicates that fermented products derived from *Lactobacillus plantarum* or corn germ meal can attenuate intestinal inflammation, enrich beneficial gut microbes, and support intestinal health, highlighting their value as functional ingredients in poultry diets [21,22].

As an edible medicinal fungus, *G. lucidum* produces lignocellulose-degrading enzymes and bioactive metabolites capable of modifying plant substrates. Fermentation with *G. lucidum* can enhance nutritional composition, functional compounds, and antioxidant activity [23,24,25], and fermented green tea has been shown to reduce lipid accumulation in obesity models [26]. Prior work also reveals that *G. lucidum* fermentation increases DNJ, polysaccharides, flavonoids, and alkaloids in mulberry leaves, substantially improving their α-glucosidase inhibitory capacity [27,28]. Despite these biochemical improvements, the incorporation of *G. lucidum*-fermented mulberry leaves into laying-hen diets has not been investigated. Despite the nutritional benefits of microbial fermentation, mulberry leaves fermented with *G. lucidum* have not been thoroughly evaluated as poultry feed. We hypothesized that *G. lucidum* fermentation would reduce antinutritional factors and improve amino acid composition. These alterations may help support antioxidant status, immunity, lipid metabolic processes, and egg quality in aged laying hens. We therefore characterized fermentation-induced nutritional changes and evaluated the effects of a 3% dietary inclusion, a level previously reported in the literature as effective, on laying performance, egg quality, and selected serum biochemical profiles.

## 2. Materials and Methods

### 2.1. Materials

Strains of *G. lucidum* (ACCC 50695) were obtained from the Agricultural Culture Collection of China (ACCC, Beijing, China). Mulberry leaves (*Morus alba* L.) were harvested from Yingzi Town, Botou City, Hebei Province, China, in September 2024. Tannin standards were obtained from Maclin (Shanghai, China), and all reagents used were of analytical grade.

### 2.2. Sample Preparation

Mulberry leaves were fermented according to the protocol of Wang et al. [29], with slight adaptations of the method described. Fresh mulberry leaves were oven-desiccated at 60 °C under forced-air circulation until mass stabilization and subsequently ground into powder. For solid-state fermentation (SSF), dried mulberry leaf powder was uniformly mixed with sterile distilled water (1:1.2, *w*/*v*), transferred into sterile edible-fungi cultivation bags (200 g/bag; 15 cm × 25 cm), and autoclaved at 121 °C for 1 h. After cooling to ambient temperature, under aseptic conditions, each bag was inoculated with two 25-mm^2^ PDA agar plugs colonized by *G-lucidum* mycelium. Inoculated substrate bags were incubated in darkness at 25 °C and 75% relative humidity until full mycelial colonization was achieved (approximately 30 days). Upon completion of incubation, fermented mulberry leaves were harvested and subjected to quality assessment, confirming absence of off-odors and visible contamination by molds or other undesirable microorganisms. Fermented substrates were first dried at 60 °C until mass stabilization and subsequently milled and sieved through a 60-mesh stainless-steel sieve.

### 2.3. Chemical Analyses

#### 2.3.1. Proximate Composition

Crude protein (CP) was analyzed following GB/T 6432-2018 [30] using the Kjeldahl procedure. Ether extract (EE) was determined according to GB/T 6433-2025 [31] by Soxhlet extraction with petroleum ether (boiling range: 30–60 °C). Moisture content was determined gravimetrically according to GB/T 6435-2014 (105 °C, 4 h) [32]. Crude ash content was determined by muffle furnace incineration at 550 °C for 6 h, following GB/T 6438-2025 [33]. All analyses were conducted in triplicate.

#### 2.3.2. Tannin and Phytic Acid Contents

Condensed tannin content was determined using GB/T 27985-2011 [34]. The standard curve for tannin quantification was y = 0.0634x + 0.051 (R^2^ = 0.9993), with a linear detection range of 0–6 mg/L.

Phytic acid was determined spectrophotometrically using a commercial kit (BC5845, Solarbio, Beijing, China) according to the manufacturer’s guidelines. The assay is based on the hydrolysis of sodium phytate (inositol hexaphosphate dodecyl sodium) by phytase under specific environmental conditions, releasing inorganic phosphorus and inositol derivatives. Under acidic conditions, the liberated inorganic phosphorus reacts with ammonium molybdate in the color-developing reagent to form a blue molybdenum blue complex, which exhibits a characteristic absorption peak at 660 nm. By quantifying the amount of inorganic phosphorus produced, the phytate content in the sample can be calculated. The assay has a detection limit of 3.5 nmol/L and a linear range of 4–200 nmol/L.

#### 2.3.3. Amino Acid Analysis

Amino acid composition was measured based on acid and alkaline hydrolysis [35]. For the quantification of most amino acids (excluding tryptophan), 50 mg of dried mulberry leaf powder was hydrolyzed with 1 mL of 6 M HCl under nitrogen at 110 °C for 24 h. For tryptophan determination, a separate alkaline hydrolysis was performed: 50 mg of sample was treated with 1 mL of 5 M NaOH and incubated at 110 °C for 22 h under nitrogen. After cooling, hydrolysates were adjusted to pH 6.5–7.0 using 6 M HCl or 5 M HClO_4_, as required, followed by filtration through a 0.22-µm nylon membrane. The filtrates were subsequently derivatized with ortho-phthalaldehyde (OPA) and 9-fluorenylmethyl chloroformate (FMOC) before UPLC-HRMS analysis. UPLC conditions: sample extracts were analyzed using a Vanquish UPLC system coupled to a Q Exactive Orbitrap mass spectrometer (Thermo Fisher Scientific Inc., Delaware, DE, USA). Separation was performed on a Waters ACQUITY UPLC BEH C18 column (1.7 µm, 50 × 2.1 mm) (Waters Corporation, Delaware, DE, USA) at 55 °C. The mobile phase was delivered at 0.5 mL/min, and the injection volume was maintained at 1 µL. The mobile phases were water with 0.1% formic acid (A) and acetonitrile with 0.1% formic acid (B). The gradient was 5% B at 0 min, 10% B at 5.5 min, 25% B at 7.5 min, 60% B at 8.0 min, 5% B at 8.5 min, and 5% B at 13.0 min.

LC-MS analysis: HRMS data were collected on a Q Exactive hybrid Q-Orbitrap mass spectrometer (Thermo Fisher Scientific Inc., Delaware, DE, USA) equipped with a heated electrospray ionization source, operating in full-MS mode. The ESI source settings were: spray voltage, 3 kV; sheath gas, 40 arb; auxiliary gas, 10 arb; sweep gas, 0 arb; capillary temperature, 320 °C; and auxiliary gas heater temperature, 350 °C. Data were acquired using Xcalibur 4.1 and processed with TraceFinder 4.1 Clinical.

### 2.4. Experimental Design, Diets, and Management

One hundred and fifty healthy Hy-Line Brown laying hens (60 weeks old) were weighed individually and apportioned randomly into three dietary regimens, each with five pens of 10 hens. The treatments were: Con (basal diet), UML (basal diet + 3% unfermented mulberry leaf powder), and FML (basal diet + 3% fermented mulberry leaf powder) [9,36]. Birds were reared at 18–20 °C under a 16L:8D photoperiod regimen. Feed was supplied twice daily at 125 g per hen per day, and water was available ad libitum. The basal diet met the Chinese Feeding Standard of Chicken [37] (Table 1). The Animal Care and Use Committee of Hebei University approved all procedures (HBUM2025079).

### 2.5. Productive Performance, Egg Quality, and Blood Sampling

Eggs were collected and evaluated on days 30 and 60. Outcomes included laying rate, average egg weight, egg-shape index, eggshell thickness, eggshell strength, yolk ratio, yolk colour, Haugh unit, albumen protein content, and yolk cholesterol. All egg quality evaluations and laboratory analyses were carried out by investigators blinded to treatment groups to avoid subjective bias. At the end of the 60-day trial, ten hens per treatment (two hens per replicate pen) were selected for blood collection. Blood was collected from the wing vein into non-heparinized tubes, allowed to clot for 30 min at room temperature, and centrifuged at 3000× *g* for 15 min at 4 °C. Serum was aliquoted and stored at −80 °C.

### 2.6. Serum Analysis

Serum T-AOC, SOD, CAT, GSH-Px, and MDA concentrations were determined spectrophotometrically using proprietary assay kits produced by Nanjing Jiancheng Bioengineering Institute (Nanjing, China) as per the supplier’s recommended procedure. Serum TP, ALB, TC, TG, HDL-C, and LDL-C were quantified using commercial assay kits (BIOSINO BIO-TEC, Beijing, China). The serum concentrations of IgA, IgG, and IgM were assessed with commercial ELISA kits (JINGMEI BIOTECHNOLOGY, Yancheng, China).

### 2.7. Statistical Analysis

Results are shown as mean ± SD unless noted otherwise. Chemical composition data for UML and FML were compared using independent-samples *t*-tests. Treatment effects on laying performance, egg quality, and serum biochemical indices were analyzed by ANOVA with Tukey’s post hoc test. Laying performance was evaluated per pen, whereas egg quality and serum biochemical variables were assessed per hen. Shapiro–Wilk and Levene tests were applied to confirm normality and homogeneity of variance prior to ANOVA. Differences were regarded as statistically significant when *p* < 0.05. All data processing and statistical analyses were executed using SPSS software (version 26.0; IBM Corp., Armonk, NY, USA).

## 3. Results

### 3.1. Proximate Composition

Table 2 summarizes the chemical profile of mulberry leaves before and after SSF with *G. lucidum*. Crude protein, moisture, and crude ash contents were significantly elevated by fermentation (*p* < 0.05), with crude fat exhibiting no significant variation. The protein content increased from 20.25 ± 0.21% in UML to 24.20 ± 0.14% in FML. Moisture content also rose from 5.96 ± 0.09% to 7.71 ± 0.19%, and ash content increased from 16.32 ± 0.08% to 20.97 ± 0.32%. Relative to UML, the protein, moisture, and ash contents in FML were elevated by 19.51%, 29.36%, and 28.49%, respectively.

### 3.2. Tannin and Phytic Acid Contents

**Figure 1:**
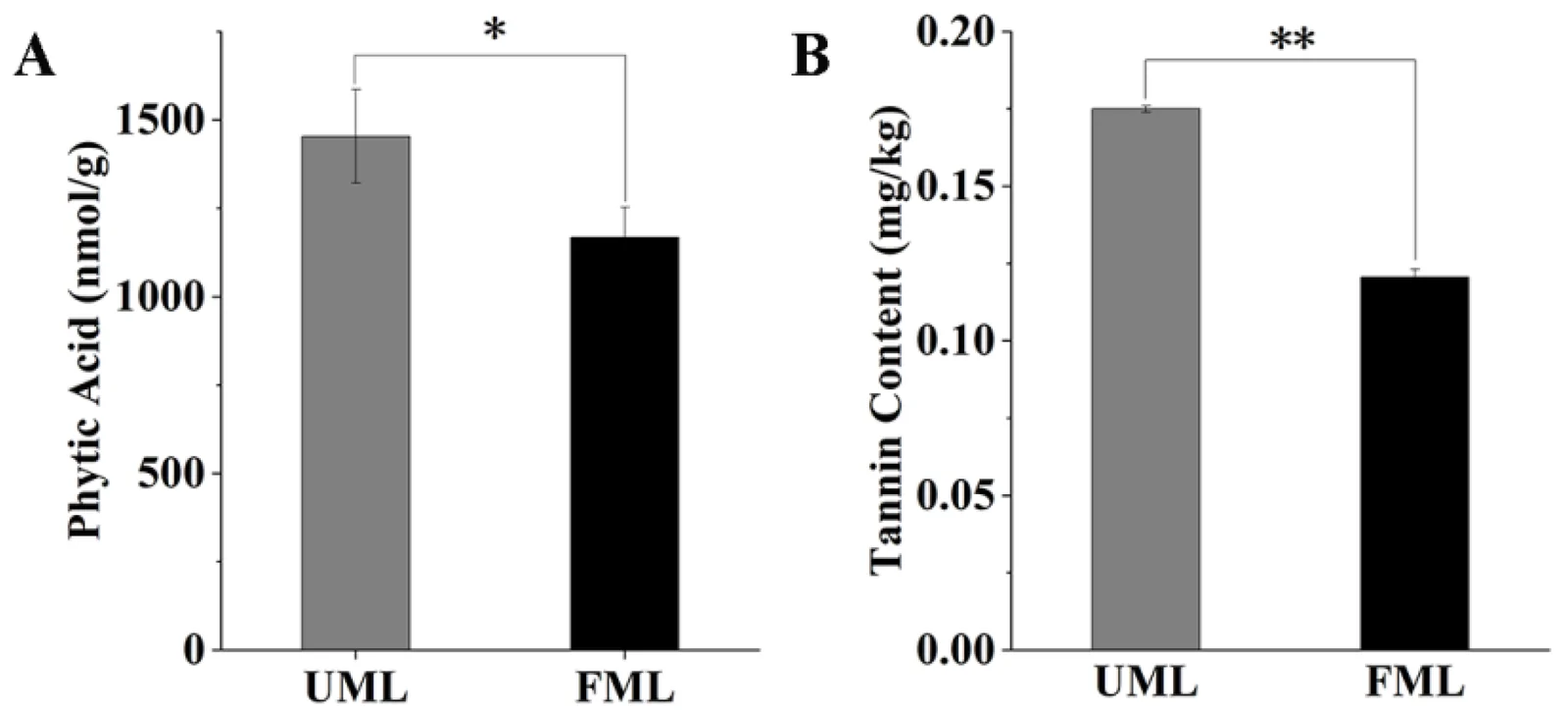
Phytic acid and tannin contents of UML and FML. (**A**) Phytic acid content and (**B**) tannin content. Data are summarized as mean ± SD. Significance levels are marked with asterisks, with * *p* < 0.05 and ** *p* < 0.01. *n* = 3 replicates per treatment. Data are expressed on a dry-matter basis.

Before fermentation, mulberry leaves contained 1453.90 ± 131.81 nmol/g phytic acid and 0.18 ± 0.01 mg/kg tannins (Figure 1). After fermentation, the corresponding values were 1168.06 ± 85.31 nmol/g and 0.12 ± 0.01 mg/kg, representing reductions of 19.61% (*p* = 0.017) and 31.03% (*p* < 0.01), respectively. Thus, *G. lucidum* fermentation reduced both measured antinutritional factors.

### 3.3. Amino Acid Analysis

The amino acid profiles before and after fermentation are shown in Table 3. Among all measured amino acids, aspartic acid, cysteine, and proline showed no significant change, while glutamic acid decreased by 10.69%. The contents of all other amino acids increased significantly following fermentation. Methionine increased from 0.042 to 0.20 g/100 g, a 4.76-fold increase. Total amino acids, essential amino acids, flavour-associated amino acids, and branched-chain amino acids increased by 12.70%, 20.41%, 5.67%, and 8.82%, respectively.

### 3.4. Effects of UML and FML on Laying Performance and Egg Quality

Most production and egg-quality variables were unaffected by UML or FML, including laying rate, egg-shape index, eggshell thickness, yolk ratio, yolk colour, albumen protein content, and Haugh unit (*p* > 0.05; Table 4). At day 60, average egg weight was lower in UML than in FML, whereas FML did not differ from Con. Both UML and FML reduced egg-yolk cholesterol relative to Con, with the largest reduction in FML. Cholesterol decreased by 24.98% and 32.02% in UML and FML, respectively, on day 30, and by 30.18% and 32.68% on day 60.

### 3.5. Effects of Dietary UML and FML on Serum Biochemical Indices

**Figure 2:**
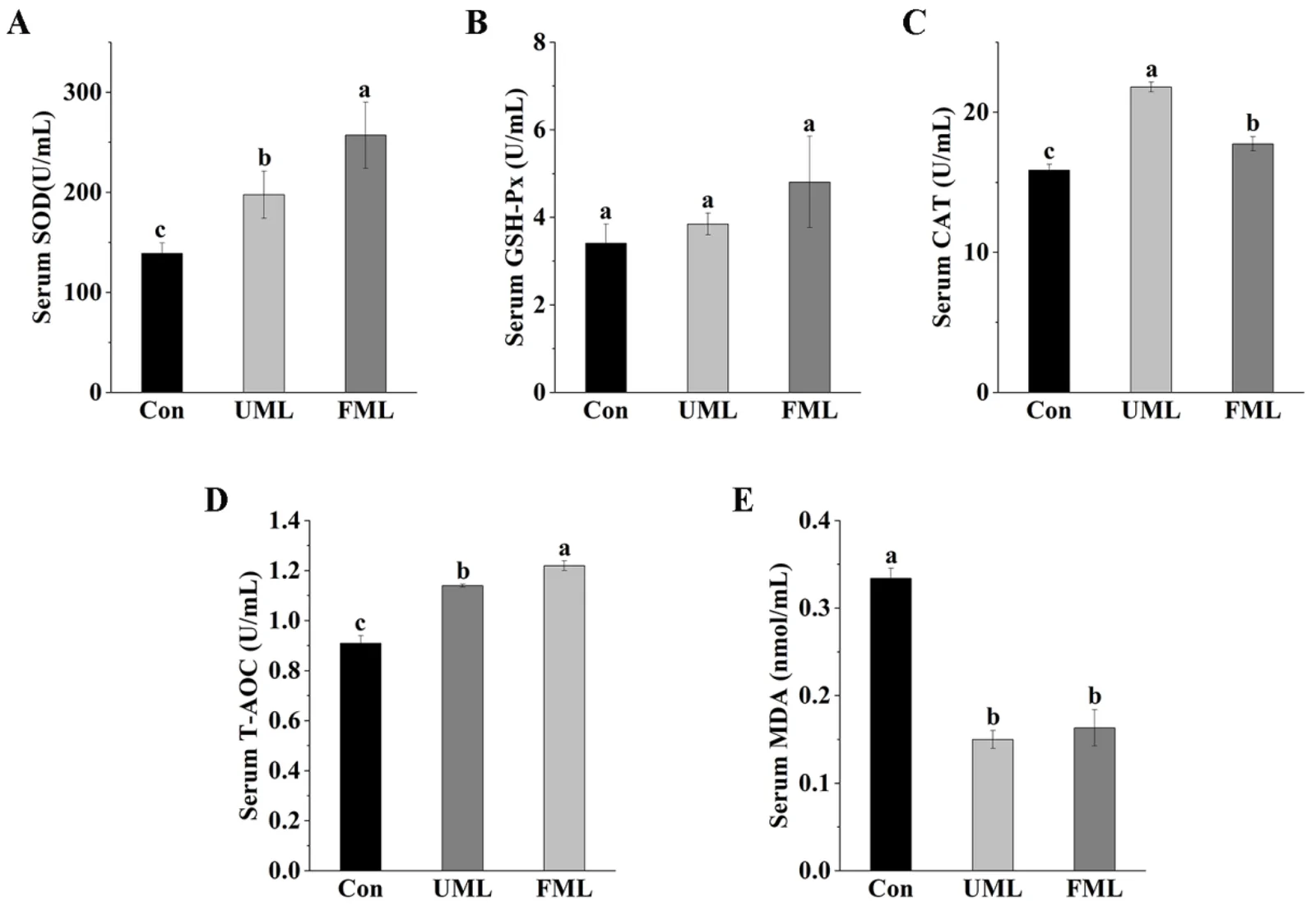
Effects of dietary UML and FML on serum antioxidant status in Hy-Line Brown laying hens. (**A**) SOD activity. (**B**) GSH-Px activity. (**C**) CAT activity. (**D**) T-AOC. (**E**) MDA concentration. Data are summarized as mean ± SD. Group-wise significant differences are marked by differing superscript letters (*p* < 0.05). *n* = 10 replicates per treatment.

Dietary UML and FML improved several serum antioxidant indices (Figure 2). SOD activity increased significantly, rising from 139.13 ± 10.30 U/mL in the Con group to 197.74 ± 23.58 U/mL in UML and further to 257.20 ± 33.03 U/mL in FML, corresponding to 42.13% (*p* < 0.01) and 84.86% (*p* < 0.01) increases, respectively. CAT activity was also elevated, increasing from 15.87 ± 0.42 U/mL in the Con group to 21.81 ± 0.35 U/mL in UML and 17.75 ± 0.51 U/mL in FML, representing significant increases of 37.81% (*p* < 0.01) and 11.85% (*p* < 0.01). T-AOC showed a significant rise from 0.91 ± 0.03 U/mL in the Con group to 1.14 ± 0.01 U/mL in UML and 1.22 ± 0.06 U/mL in FML, increasing by 25.27% (*p* < 0.01) and 36.06% (*p* < 0.01), respectively. In contrast, MDA content decreased markedly, dropping from 0.33 ± 0.01 nmol/mL in the Con group to 0.15 ± 0.01 nmol/mL in UML and 0.16 ± 0.02 nmol/mL in FML, reflecting significant reductions of 54.54% (*p* < 0.01) and 51.52% (*p* < 0.01).

**Figure 3:**
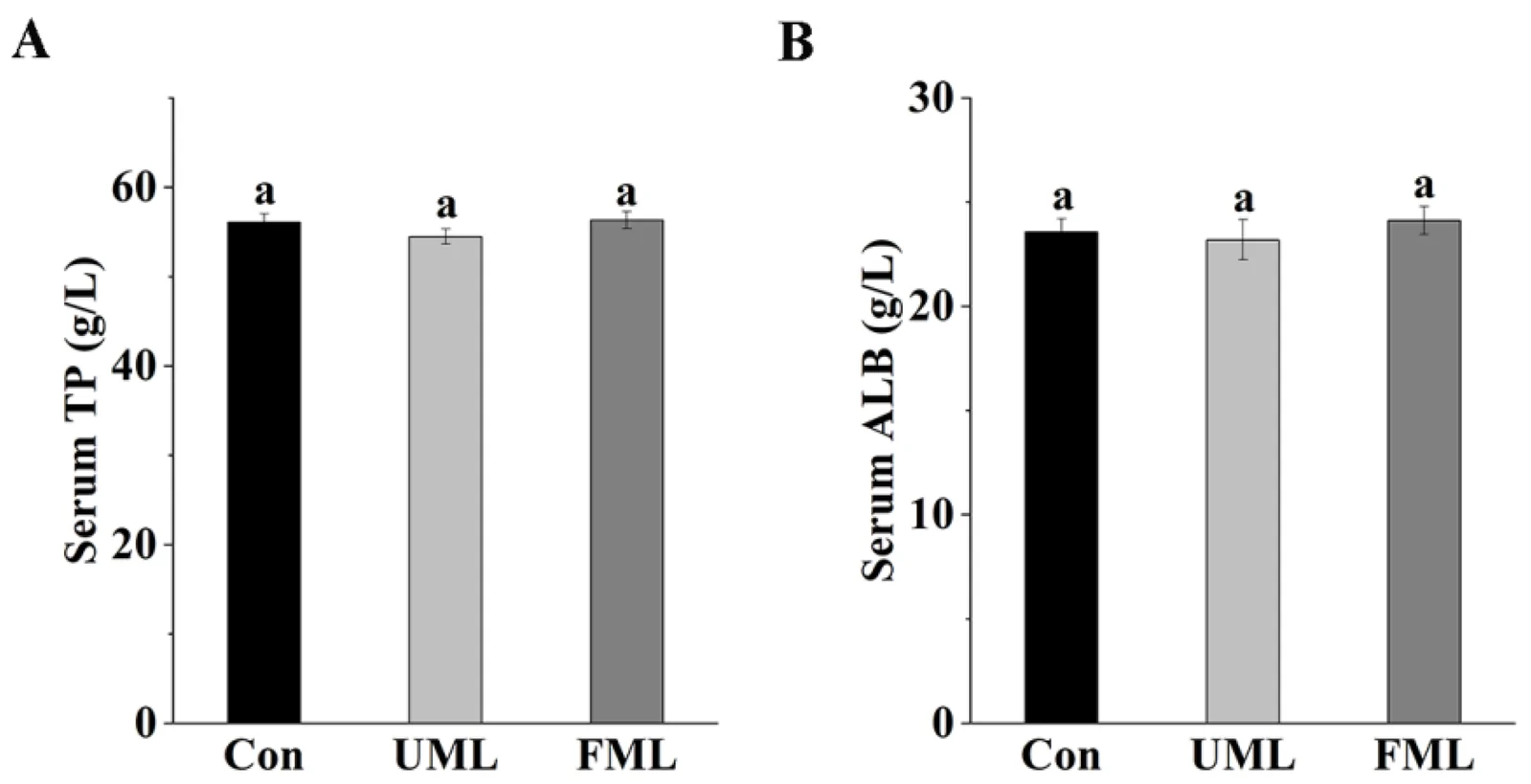
Effects of dietary UML and FML on serum protein indices in Hy-Line Brown laying hens. (**A**) Serum TP concentration. (**B**) Serum ALB concentration. Data are summarized as mean ± SD. Values with the superscript “a” indicate no significant difference among groups (*p* > 0.05). *n* = 10 replicates per treatment.

Serum protein indices are shown in Figure 3. Total protein concentrations were 56.08 ± 0.96, 54.50 ± 0.85, and 56.35 ± 0.94 g/L in Con, UML, and FML, respectively. Albumin concentrations were 23.57 ± 0.64, 23.20 ± 0.96, and 24.12 ± 0.67 g/L, respectively. Neither UML nor FML significantly affected serum total protein or albumin (*p* > 0.05).

**Figure 4:**
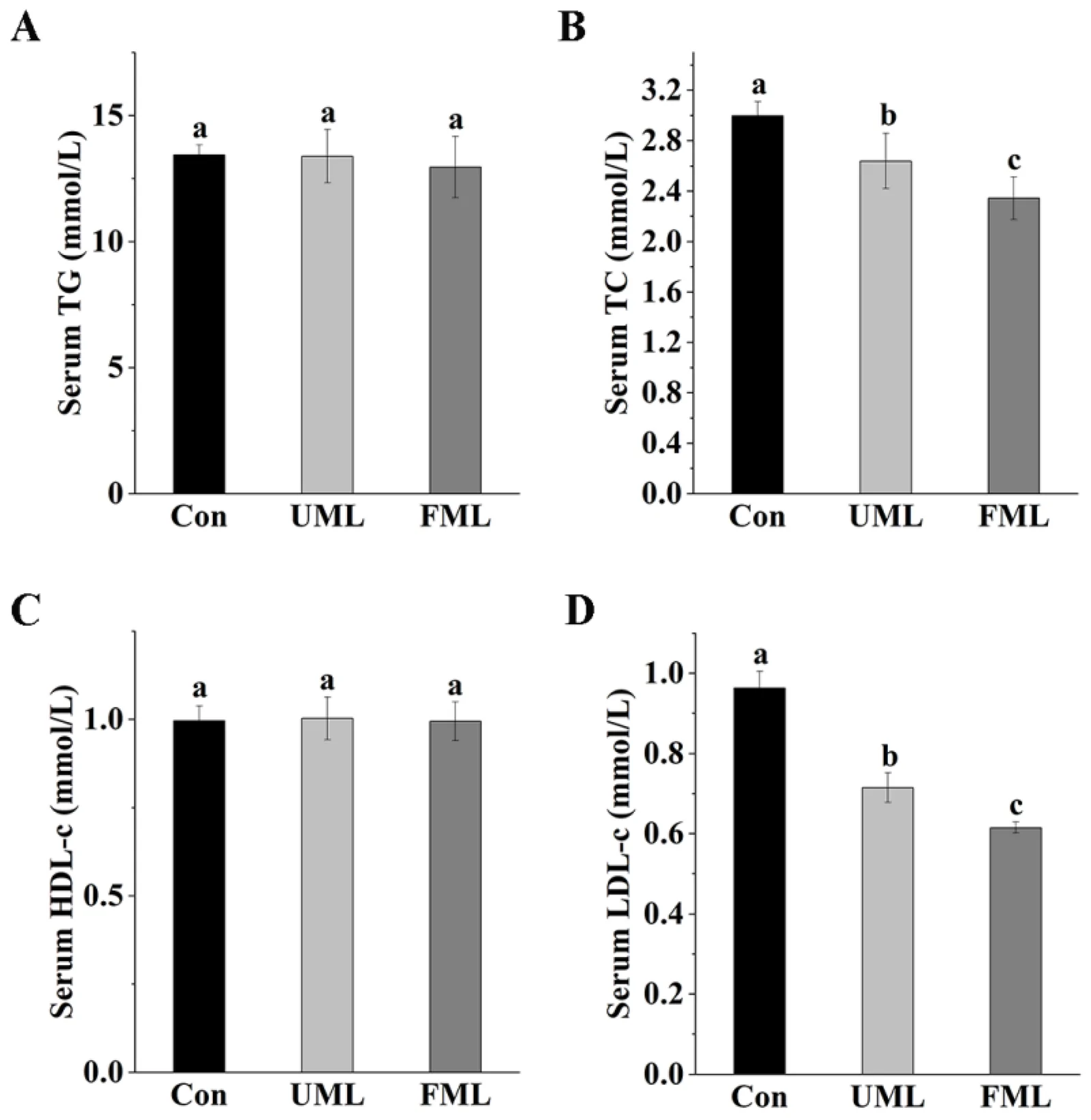
Influence of dietary UML and FML on serum lipid profiles in Hy-Line Brown laying hens. (**A**) TG. (**B**) TC. (**C**) HDL-C. (**D**) LDL-C. Data are summarized as mean ± SD. Group-wise significant differences are marked by differing superscript letters (*p* < 0.05). *n* = 10 replicates per treatment.

Serum lipid indices are shown in Figure 4. Neither UML nor FML significantly affected triglyceride or HDL-C concentrations. TC levels decreased significantly, falling from 3.00 ± 0.11 mmol/L in the Con group to 2.64 ± 0.22 mmol/L in UML and 2.35 ± 0.17 mmol/L in FML, corresponding to reductions of 12.00% (*p* = 0.002) and 21.67% (*p* < 0.001), respectively. Similarly, LDL-C declined markedly, dropping from 0.96 ± 0.04 mmol/L in the Con group to 0.72 ± 0.04 mmol/L in UML and 0.62 ± 0.01 mmol/L in FML, representing significant decreases of 25.00% (*p* < 0.001) and 35.42% (*p* < 0.001).

**Figure 5:**
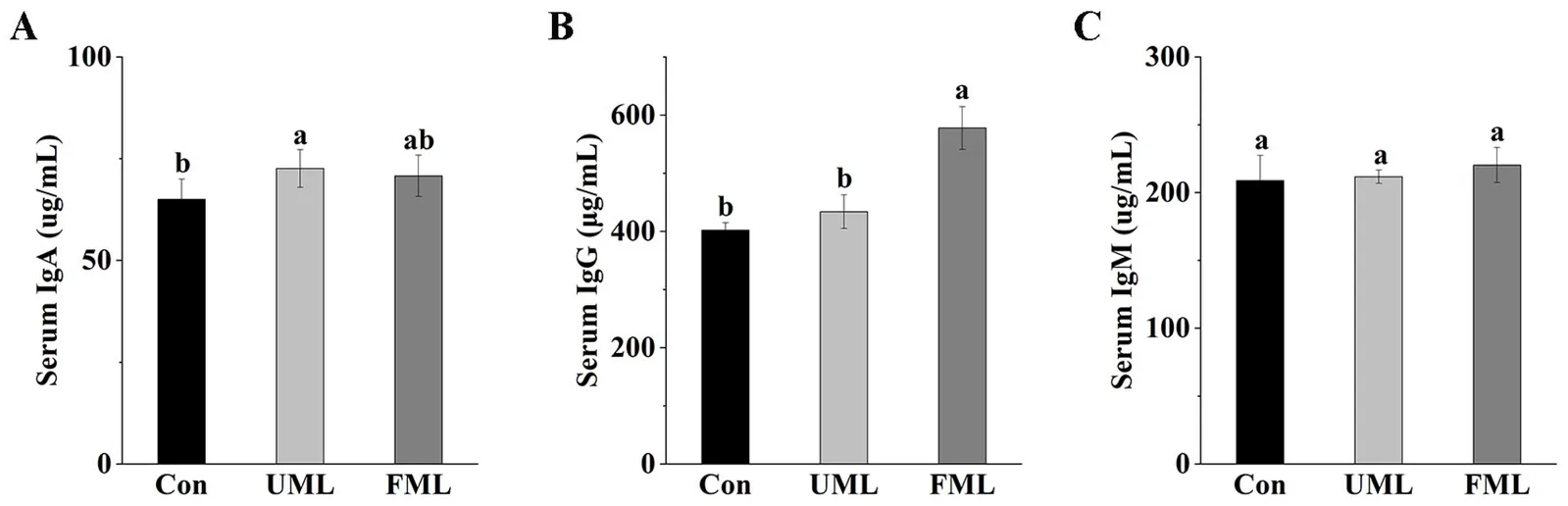
Effects of dietary UML and FML on serum immunoglobulin indices in Hy-Line Brown laying hens. (**A**) Serum IgA concentration. (**B**) Serum IgG concentration. (**C**) Serum IgM concentration. Data are summarized as mean ± SD. Group-wise significant differences are marked by differing superscript letters (*p* < 0.05). *n* = 10 replicates per treatment.

Serum immunoglobulin concentrations are shown in Figure 5. IgA concentration increased from 65.06 ± 4.90 μg/mL in the Con group to 72.61 ± 0.22 μg/mL in the UML group, representing an 11.60% (*p* = 0.017) increase, while the FML group showed no significant difference compared with the Con. In contrast, IgG levels rose markedly, increasing from 402.31 ± 12.78 μg/mL in the Con group to 577.91 ± 36.68 μg/mL in the FML group, a significant elevation of 43.65% (*p* < 0.001). Neither treatment significantly affected IgM.

## 4. Discussion

### 4.1. Proximate Composition

Fermentation with *G. lucidum* led to higher protein, moisture, and ash levels in mulberry leaves. The increase in protein content may reflect losses of carbon and dry matter during fermentation. *G. lucidum* utilizes mulberry-leaf proteins to support mycelial growth and biomass synthesis [18,24]. In addition, fungal colonization can alter substrate structure and water-holding capacity, which may contribute to the higher moisture content observed in fermented leaves [38].

### 4.2. Tannin and Phytic Acid Contents

Phytic acid and tannins constitute major antinutritional factors in plant-derived feed materials. Phytate is poorly available to monogastric animals and can chelate minerals or hinder nutrient utilization [13,39,40]. Tannins likewise reduce protein digestibility by forming complexes with proteins and digestive enzymes [14]. Our observations agree with studies showing that microbial and fungal fermentation lowers antinutritional factors in plant substrates [16,17,18].

### 4.3. Amino Acid Analysis

Fermentation with *G. lucidum* increased the total, essential, and branched-chain amino acid contents of mulberry leaves. Methionine is limiting in many poultry diets and contributes to protein synthesis, methyl-group metabolism, antioxidant defence, and immune function [40]. Branched-chain amino acids also have important metabolic and nutritional functions [41,42,43]. The decrease in Glu content may be attributed to its utilization by the mycelia during the growth of *Ganoderma lucidum*, whereas the non-significant increases in Asp and Pro likely reflect their limited synthesis during metabolic activities [38].

### 4.4. Effects of UML and FML on Laying Performance and Egg Quality

Neither UML nor FML affected laying rate or most conventional egg-quality parameters at the 3% inclusion level. Both treatments lowered yolk cholesterol, with FML producing a significantly greater reduction than UML. These observations corroborate earlier evidence that mulberry leaf products can regulate lipid metabolism and egg-quality parameters in laying hens [11,12]. Comparable cholesterol-lowering effects have also been documented for other plant-derived or fermented dietary additives [44]. Although the present study demonstrates efficacy at a single inclusion level, it does not establish the optimal dose.

### 4.5. Effects of Dietary UML and FML on Serum Biochemical Parameters

Both UML and FML increased serum SOD and CAT activities and reduced MDA levels, and FML produced a greater enhancement in T-AOC, whereas GSH-Px remained unchanged. These results indicate that both unfermented and fermented mulberry leaves can improve serum antioxidant enzyme activities in laying hens. Earlier research has indicated that mulberry leaves and their derivatives enhance antioxidant capacity in poultry, likely attributable to their flavonoid, polysaccharide, and polyphenol constituents [6,10,12,45]. Fermentation with *G. lucidum* modifies phenolic compounds, isoflavones, polysaccharide activity, and antioxidant capacity in plant substrates [46,47]; in mulberry leaves, it increases flavonoids and polyphenols, enhances hypoglycemic activity, and converts insoluble dietary fiber into soluble forms, thereby enriching functional components and contributing to the greater improvement in serum antioxidant enzyme activities observed in the FML group [27,28]. Neither UML nor FML significantly altered serum triglyceride or HDL-C concentrations. Both treatments reduced TC and LDL-C relative to Con, and the FML group showed a significantly greater reduction than the UML group. These findings agree with reports that mulberry leaf products can improve lipid-related outcomes in laying hens [11,48] and experimental models [8]. Fermentation-induced metabolites in other plant substrates may interact with lipid- and redox-related targets [46]. The mechanisms underlying the observed changes in serum lipid profiles require further elucidation.

Compared with Con, UML significantly increased serum IgA levels, whereas FML significantly elevated IgG levels, indicating that both mulberry leaves and fermented mulberry leaves can modulate immunoglobulin profiles in aged laying hens. Previous studies have shown that *Ganoderma lucidum* fermentation enriches functional metabolites such as ganoderic acids and ganoderic triols in the substrate, and these compounds have been reported to modulate immune responses in poultry [24,49,50,51]. Accordingly, we speculate that the elevated IgG levels in the FML group may be linked to the accumulation of bioactive metabolites produced during fermentation. Previous studies have shown that fermented feed products can enhance immune function in chickens by increasing *IL*-*10* expression while suppressing *MAPK*-related genes such as *ERK* and *p38*. This evidence reinforces the functional value of fermented plant materials in poultry diets [21,22]. Nonetheless, the specific immunomodulatory mechanisms of fermented mulberry leaves require further investigation.

This study has several limitations. Bioactive compounds, including flavonoids, polysaccharides, and triterpenoids, were not quantified after fermentation. As a result, the mechanisms underlying the lipid, antioxidant, and immunoglobulin responses remain unresolved. Only one inclusion level was tested, preventing dose–response evaluation. In addition, the current study focuses on changes in nutritional components and does not evaluate digestive characteristics, and metabolomic analyses were not performed to characterize fermentation-derived metabolites. The study also lacked assessments of intestinal morphology, gut microbiota composition, and molecular markers related to antioxidant and immune pathways. These limitations restrict the ability to fully interpret the biological mechanisms and functional efficacy of fermented mulberry leaves.

## 5. Conclusions

*G. lucidum* fermentation improved the measured nutritional profile of mulberry leaves and reduced phytic acid and tannins. Fermentation increased crude protein and most amino acids, including methionine. With 3% dietary inclusion, no significant treatment effects were observed for laying rate or most egg-quality traits. It was associated with lower egg-yolk cholesterol, improved antioxidant enzyme activities, lower serum TC and LDL-C, and higher serum IgG. Compared with UML, FML produced significant stronger changes in selected antioxidant and lipid-related indices. These results justify further assessment of FML as a low-inclusion functional dietary ingredient for aged laying hens. The mechanisms underlying the observed changes in antioxidant status, serum lipid profiles, and immunoglobulin concentrations require further elucidation. Commercial use should await dose–response, digestibility, safety, and mechanistic studies with clearly defined experimental units and larger replication.

## Figures and Tables

**Table 1:** Nutrient composition and formulation of the basal diet.

Ingredient	Content (%)	Nutrient Level	Content (%)
Corn	61.50	Metabolizable Energy (MJ/kg)	11.37
Soybean meal	22.00	Moisture	10.00
Soybean oil	1.00	Crude Ash	12.20
Limestone	9.00	Crude Protein	16.10
Wheat bran	4.50	Crude Fat	4.00
Premix ^1^	2.00	Crude Fiber	3.60
Total	100.00	Total Phosphorus	0.39
		Calcium	3.56
		Methionine	0.37
		Lysine	0.88
		Nitrogen-Free Extract (g/100 g)	54.10

^1^ The premix provides per kilogram of diet: Vitamin A 15,000 IU, Vitamin D_3_ 3000 IU, Vitamin E 30 mg, Vitamin K 2 mg, Vitamin B_1_ 2 mg, Vitamin B_2_ 4.5 mg, Vitamin B_6_ 2.2 mg, Vitamin B_12_ 0.04 mg, Niacin 30 mg, Pantothenic Acid 6 mg, Folic Acid 0.7 mg, Biotin 0.25 mg, Iron 70 mg, Copper 8 mg, Zinc 80 mg, Iodine 0.3 mg, Manganese 90 mg. All nutrient levels are calculated values.

**Table 2:** Proximate composition of unfermented mulberry leaves (UML) and fermented mulberry leaves (FML).

Group	UML	FML	*p*-Value
Crude Protein (%)	20.25 ± 0.21	24.20 ± 0.14 **	<0.001
Moisture (%)	5.96 ± 0.09	7.71 ± 0.19 **	<0.001
Crude Ash (%)	16.32 ± 0.08	20.97 ± 0.32 **	<0.001
Crude Fat (%)	2.97 ± 0.44	2.94 ± 0.22	0.912

Data are summarized as mean ± SD. Asterisks indicate significant differences (** *p* < 0.01). *n* = 3 replicates per treatment. Values are expressed on a dry matter basis.

**Table 3:** Amino acid profiles of UML and FML.

	Amino Acids (g/100 g DM)		*p*-Value
	UML	FML	
NEAA			
Asp	1.72 ± 0.13	1.83 ± 0.11	0.153
Ser	0.82 ± 0.03	0.92 ± 0.03 *	0.010
Glu	1.59 ± 0.05	1.42 ± 0.03 *	0.004
Gly	1.31 ± 0.02	1.48 ± 0.01 **	<0.001
Ala	1.22 ± 0.03	1.40 ± 0.03 **	<0.001
Cys	0.02 ± 0.00	0.02 ± 0.00	0.405
Tyr	0.53 ± 0.01	0.57 ± 0.01 **	<0.001
Pro	1.45 ± 0.05	1.53 ± 0.05	0.060
EAA			
Phe	1.03 ± 0.02	1.14 ± 0.04 **	<0.001
His	0.35 ± 0.01	0.38 ± 0.01 **	<0.001
Ile	0.82 ± 0.01	0.90 ± 0.01 **	<0.001
Leu	1.56 ± 0.03	1.67 ± 0.03 **	<0.001
Lys	0.85 ± 0.01	1.46 ± 0.03 **	<0.001
Met	0.04 ± 0.00	0.20 ± 0.01 **	<0.001
Thr	0.94 ± 0.01	1.06 ± 0.02 **	<0.001
Trp	0.14 ± 0.00	0.14 ± 0.00 *	0.026
Val	1.02 ± 0.02	1.13 ± 0.02 **	<0.001
Arg	1.05 ± 0.07	1.28 ± 0.12 *	0.021
Cys + Met	0.06 ± 0.00	0.21 ± 0.00 **	<0.001
Phe + Tyr	1.55 ± 0.00	1.72 ± 0.00 **	<0.001
TAA	15.08 ± 0.30	16.78 ± 0.05 **	<0.001
TEAA	7.79 ± 0.10	9.38 ± 0.17 **	<0.001
FAA	8.11 ± 0.21	8.57 ± 0.12 *	0.012
BCAAs	3.40 ± 0.05	3.70 ± 0.07 **	<0.001

Data are summarized as mean ± SD. Asterisks signify the respective statistical significance levels with * *p* < 0.05 and ** *p* < 0.01. *n* = 3 replicates per treatment.

**Table 4:** Impact of mulberry leaves (UML) and fermented mulberry leaves (FML) on laying performance in Hy-Line Brown laying hens.

Items	Time (Day)	Con	UML	FML	*p*-Value
Laying rate (%)	30	75.27 ± 1.82 ^a^	74.33 ± 1.39 ^a^	75.67 ± 1.18 ^a^	0.377
	60	75.93 ± 1.98 ^a^	73.67 ± 2.49 ^a^	76.27± 2.01 ^a^	0.164
Average egg weight (g)	30	62.18 ± 2.26 ^a^	61.06 ± 3.99 ^a^	66.18 ± 7.77 ^a^	0.299
	60	60.00 ± 3.10 ^ab^	56.09 ± 2.69 ^b^	64.16 ± 2.62 ^a^	0.002
Ovality index	30	0.75 ± 0.02 ^a^	0.77 ± 0.01 ^a^	0.74 ± 0.04 ^a^	0.143
	60	0.78 ± 0.04 ^a^	0.77 ± 0.02 ^a^	0.74 ± 0.02 ^a^	0.249
Eggshell thickness (mm)	30	0.46 ± 0.02 ^a^	0.44 ± 0.04 ^a^	0.46 ± 0.01 ^a^	0.345
	60	0.44 ± 0.03 ^a^	0.42 ± 0.04 ^a^	0.45 ± 0.02 ^a^	0.316
Egg yolk ratio (%)	30	26.94 ± 2.33 ^a^	27.10 ± 0.98 ^a^	25.82 ± 1.54 ^a^	0.459
	60	28.01 ± 1.44 ^a^	28.16 ± 1.92 ^a^	27.50 ± 2.44 ^a^	0.859
Yolk color	30	7.20 ± 1.64 ^a^	7.60 ± 1.14 ^a^	6.80 ± 2.17 ^a^	0.764
	60	6.40 ± 1.52 ^a^	6.40 ± 1.14 ^a^	6.80 ± 1.79 ^a^	0.890
Eggshell strength (gf)	30	3980.15 ± 702.90 ^a^	4691.48 ± 918.92 ^a^	4034.91 ± 553.53 ^a^	0.278
	60	4068.23 ± 685.16 ^a^	4264.86 ± 898.88 ^a^	4290.73 ± 605.32 ^a^	0.875
Haugh unit	30	72.72 ± 10.95 ^a^	66.88 ± 6.87 ^a^	63.50 ± 10.78 ^a^	0.349
	60	66.08 ± 9.04 ^a^	67.79 ± 9.99 ^a^	76.44 ± 12.61 ^a^	0.294
Albumen protein content (%)	30	12.28 ± 0.05 ^a^	12.33 ± 0.15 ^a^	12.48 ± 0.14 ^a^	0.995
	60	12.38 ± 0.21 ^a^	12.41 ± 0.45 ^a^	12.39 ± 0.25 ^a^	0.190
Yolk cholesterol (mg/100 g)	30	325.03 ± 1.60 ^a^	243.83 ± 1.54 ^b^	220.97 ± 0.55 ^c^	<0.001
	60	332.00± 3.61 ^a^	231.80 ± 3.74 ^b^	223.50 ± 0.78 ^c^	<0.001

Data are summarized as mean ± SD. Significant differences within a row are represented by distinct superscript letters (*p* < 0.05). *n* = 5 replicates per treatment.

## Data Availability

The original contributions presented in this study are included in the article. Further inquiries can be directed to the corresponding authors.

## Abbreviations

ALB	Albumin
GSH-Px	Glutathione peroxidase
IgA	Immunoglobulin A
IgM	Immunoglobulin M
MDA	Malondialdehyde
T-AOC	Total antioxidant capacity
TG	Triglyceride
CAT	Catalase
HDL-c	High-density lipoprotein cholesterol
IgG	Immunoglobulin G
LDL-c	Low-density lipoprotein cholesterol
SOD	Superoxide dismutase
TC	Total cholesterol
TP	Total protein
